# Search for Natural Compounds That Increase Apolipoprotein A‐I Transcription in HepG2 Cells: Specific Attention for BRD4 Inhibitors

**DOI:** 10.1002/lipd.12204

**Published:** 2019-12-08

**Authors:** Sophie E. van der Krieken, Pieter C. van‐der Pijl, Yuguang Lin, Herman E. Popeijus, Ronald P. Mensink, Jogchum Plat

**Affiliations:** ^1^ NUTRIM School of Nutrition and Translational Research in Metabolism, Department of Nutrition and Movement Sciences Maastricht University PO Box 616, 6200 MD Maastricht The Netherlands; ^2^ Unilever Research & Development Vlaardingen Olivier van Noortlaan 120, 3133 AT Vlaardingen The Netherlands

**Keywords:** apolipoprotein A‐I, BET inhibitor, BRD4, high‐density lipoprotein, *in silico* structural similarity search, natural compounds

## Abstract

Although increasing apolipoprotein A‐I (apoA‐I) might lower the cardiovascular disease risk, knowledge on natural compounds that elevate apoA‐I transcription is limited. Therefore, the aim of this study was to discover natural compounds that increase apoA‐I transcription in HepG2 cells. Since BRD4 inhibition is known to elevate apoA‐I transcription, we focused on natural BRD4 inhibitors. For this, the literature was screened for compounds that might increase apoA‐I and or inhibit BRD4. This resulted in *list A*, (apoA‐I increasers with unknown BRD4 inhibitor capacity), *list B* (known BRD4 inhibitors that increase apoA‐I), and *list C* (BRD4 inhibitors with unknown effect on apoA‐I). These compounds were compared with the compounds in two natural compound databases. This resulted in (1) a common substructure (ethyl‐benzene) in 60% of selected BRD4‐inhibitors, and (2) four compounds that increased ApoA‐I: hesperetin, equilenin, 9(S)‐HOTrE, and cymarin. Whether these increases are regulated *via* BRD4 inhibition and the ethyl‐benzene structure inhibits BRD4 requires further study.

**Abbreviations**
ABCA1: ATP‐binding cassette A1
ADME: adsorption, distribution, metabolism, and excretion
ApoA‐I: apolipoprotein A‐I
BET inhibitor: bromodomain and extraterminal inhibitor
BRD1‐4: bromodomain‐containing protein 1, 2, 3, or 4
CSL112: apolipoprotein A‐I [human]
CVD: cardiovascular disease
DMSO: dimethylsulfoxide
DSM: Dutch State Mines
ER‐stress: endoplasmic reticulum stress
FCFP4: functional‐class fingerprints 4
HaCaT: human skin keratinocyte cell line
HDL: high‐density lipoprotein
HepG2: human hepatocellular liver carcinoma
IC_50_: half‐maximal inhibitory concentration
JNK: c‐Jun N‐terminal kinase
MEM: minimum essential medium
NEAA: nonessential amino acids
NIH3T3: National Institutes of Health 3‐day transfer, inoculum 3 × 10^5^ mouse fibroblast cells.
NWO: Netherlands Organization for Scientific Research
SHIME: simulator of the human intestinal microbial ecosystem
STW: Dutch Technology Foundation

## Introduction

Cholesterol efflux capacity is defined as the amount of cholesterol taken up from cholesterol‐loaded macrophages by high‐density lipoprotein (HDL) particles. It is inversely associated with the incidence of cardiovascular events (Rohatgi et al., 2014). As an elevated *in vitro* cholesterol efflux capacity may reflect increased reverse cholesterol transport *in vivo*; the efflux capacity may be a useful parameter for the development of cardiovascular disease (CVD)‐lowering strategies (Rohatgi et al., 2014). Apolipoprotein A‐I (apoA‐I) is the principal component of HDL, which can take up cholesterol by binding to the ATP‐binding cassette A1 (ABCA1), the trans membrane cholesterol transporter on macrophages (Phillips, 2014). The plasma concentration of apoA‐I is associated with increased cholesterol efflux capacity (Saleheen et al., 2015). Therefore, a promising strategy to increase cholesterol efflux capacity is to increase the amount of nascent HDL particles by increasing *de novo* apoA‐I production (Dullens et al., 2007; Smits et al., 2014). The effectiveness of increasing apoA‐I concentrations in the combat against CVD is supported by several *in vivo* animal (Rubin et al., 1991; Schultz et al., 1993) and human studies (Nissen et al., 2003; Tricoci et al., 2015). For example, intravenous infusion of recombinant apoA‐I particles decreased atherosclerosis progression, as it reduced the atheroma volume in patients with acute coronary syndromes (Nissen et al., 2003). Moreover, the use of apoA‐I mimetics like CSL112 (Tricoci et al., 2015) clearly enhanced cholesterol efflux capacity. Besides the involvement of apoA‐I in enhancing cholesterol efflux capacity, apoA‐I may also provide other cardioprotective effects. ApoA‐I is antiinflammatory (Umemoto et al., 2013), antithrombotic (Epand et al., 1994), and has glucose‐lowering properties (Dalla‐Riva et al., 2013; Drew et al., 2009). Altogether, this illustrates the crucial role for elevating apoA‐I production in CVD risk management.

In addition, studies have indicated a positive role for the family of bromodomain and extra‐terminal (BET) protein inhibitors to increase apoA‐I production. For example, in *in vitro* as well as in *in vivo* studies, the BET inhibitor RVX208 (or apabetalone) increased apoA‐I transcription and protein production (Gilham et al., 2016). Additionally, there are many other compounds with BET‐inhibiting function and the capacity to increase apoA‐I synthesis, at least *in vitro*, such as JQ1(+) (Kempen et al., 2013), Ro11‐1464 (Zanotti et al., 2011), GW841819X (Chung et al., 2011), GSK1210151A or I‐BET151 (Seal et al., 2012), alaprazolam (Filippakopoulos et al., 2012), GSK1324762A or I‐BET762 (Mirguet et al., 2012), and thieno‐ or benzo‐triazolodiazepines (Kempen et al., 2013) such as U‐34599 and U‐51477 (Princen JMG May 28; Princen and Kooistra, 2003; Kempen et al., 2013). In humans, four types of BET proteins have been identified, namely, bromodomain‐containing protein (BRD) 2, BRD3, BRD4, and testes‐specific BRDT. Although many BET inhibitors are multi‐BET active, *in vitro* experiments have shown that specifically the silencing of BRD4 is involved in increasing apoA‐I production (Chung et al., 2011). For example, JQ1(+) and RVX208 inhibit BRD4, which may explain their effects on increasing apoA‐I production. Currently, BET‐inhibition is considered a promising route to increase apoA‐I transcription and most BET inhibitors under development are of synthetic origin. Possibly, natural compounds can—assuming they pass safety assessment, affordable sourcing, and have favorable ADME properties—be used as a functional food ingredient. Therefore, the aim of this study was to identify natural compounds that increase apoA‐I transcription, by an *in silico* and *in vitro* approach based on a literature review. Specific attention was paid to the role of BRD4 inhibition.

## Materials and Methods

### General Approach

**Figure 1:**
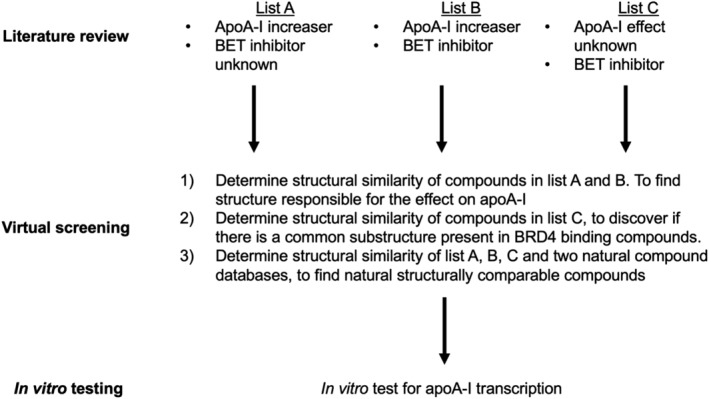
Study design. In order to find natural compounds with the ability to increase apoA‐I transcription in HepG2 cells, literature was reviewed. This resulted in lists A, B, and C. Molecules from those lists were compared with those of two databases of natural compounds. One of the lists (C) contained many hits, allowing a common substructure search. Finally, four hits from the screening, and for each compound a structurally comparable compound, were tested for their effect on apoA‐I transcription in HepG2 cells

To identify new, natural compounds, or functional (sub‐)structures that increase apoA‐I transcription, three lists (Lists A, B, and C) were compiled based on a literature review, and via a database of bioactivities (Gaulton [22]). The compounds in these lists were compared with those from two databases containing natural compounds: a company‐owned database and a commercially available one. Next, most similar compounds were tested *in vitro* for their ability to increase apoA‐I transcription. For a schematic representation of the study design, see Fig. 1.

### Literature Review

To identify known natural compounds that increase apoA‐I production or HDL, the literature was scrutinized using PubMed for articles published until August 2015. As search term (((BRD*) OR bromodomain)) AND (((apo*) OR High‐Density Lipoproteins, Pre‐beta) OR apolipoprotein A‐I) was used. In addition, a well‐curated database of bioactivities (Gaulton et al., 2017) was used to identify natural compounds and synthetic compounds, which inhibit BRD4. Additionally, we recently identified cymarin and 9(S)‐HOTrE as apoA‐I transcriptional elevating compounds (van der Krieken et al., 2018). Therefore, these two compounds were included in our literature review results as well.

This resulted in the formation of three lists (Fig. 1): *List A* contained compounds that increase apoA‐I production with unknown BRD4 inhibiting capacity. *List B c*ontained compounds that were BRD4 inhibitors and increased apoA‐I production. *List C* was composed of known BRD4 inhibitors, with unknown effects on apoA‐I production. Only BRD4 inhibitors with IC_50_ values <500 nM were selected.

### *In Silico* Screening for Natural Ingredients with Potential to Increase apoA‐I

Two databases were searched for natural compounds that may increase apoA‐I transcription: a database provided by DSM (DSM, Delft, The Netherlands) containing 2000 natural compounds) and a commercially available one, the Dictionary of Natural Products (version 18.1; Francis & Taylor) containing about 260.000 natural compounds.

Structural similarities between compounds identified from the literature and molecules present in those databases were determined using the Tanimoto algorithm based on circular fingerprints of four atoms (FCFP4). This was performed for molecules from each list.

The method used to screen for molecules depended on the list of molecules: since lists A and B contained relatively few compounds, a structural similarity search was performed for each compound from these lists in the aforementioned natural product databases *via* Tanimoto‐based similarities. In addition, the structural similarity of compounds from lists A and B was shown in a structural similarity matrix. By performing a substructure search in list C, we determined if the BRD4 inhibitors in this list contain a common substructure.

In addition, retrieved compounds were filtered on having a structural similarity of at least 0.5, and being commercially available.

Finally, BioVIA's PipelinePilot version 9.2 was used for all cheminformatic calculations (determination of structural similarity and identification of a common substructure).

### In Vitro apoA‐I Transcription

Human hepatocellular liver carcinoma (HepG2) cells (kindly provided by S. Braesch‐Andersen, Mabtech, Nacka Strand, Sweden) were cultured at 37°C in a humidified atmosphere and 5% CO_2_. For cell culturing, minimum essential medium (MEM) was used supplemented with 10% fetal calf serum (v/v, South‐American, Greiner Bio‐one, Frickenhausen, Germany), L‐glutamine (Invitrogen Life Technologies, Carlsbad, CA, USA), 1% penicillin/streptomycin (v/v), 1% nonessential amino acids (NEAA, v/v), and 1% sodium pyruvate (v/v, all from Invitrogen Life Technologies). To gain insight into the effect of the selected natural compounds on apoA‐I transcription, HepG2 cells were exposed for 48 h to different doses of each compound. Stocks were prepared in recommended carrier solutions and were diluted in culture medium. For carrier controls, maximally 0.5% DMSO or ethanol was used. After incubation, cells were microscopically inspected and pictures of each condition were made to confirm cell vitality (data not shown). Different doses of the BRD4 inhibitor RVX208 (Bioconnect, Huissen, The Netherlands) were used as positive controls for their ability to increase apoA‐I transcription. Furthermore, in each experiment 3 μM JQ1(+) (Tocris Bioscience, Abingdon, UK), another known BRD4 inhibitor was used as a control for increased apoA‐I transcription in HepG2 cells.

### ApoA‐I qPCR Measurement

Total RNA was isolated according to the Qiagen Trizol protocol. Next, cDNA was produced using Taqman reagents. ApoA‐I (Hs00163641_m1) and internal control cyclophilin A (Hs99999904_m1) mRNA expression was determined using the 7300 Real‐Time PCR System. Both TaqMan Gene Expression Assays and reagents were obtained from Applied Biosystems (Warrington, UK).

## Results

### Literature‐Derived ApoA‐I Increasing Compounds and/or BRD4 Inhibitors

**Figure 2:**
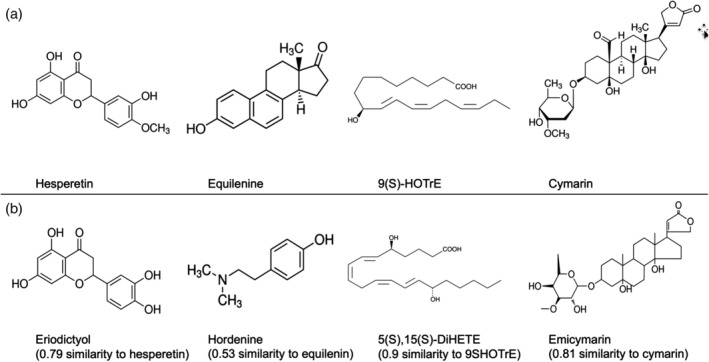
Comparison of lists A and B to the DNP and DSM natural databases resulted in the confirmation of four structures: hesperetin, equilenin, (9)S‐HOTrE, and cymarin (a). In (b), the structurally comparable compounds are presented: eriodictyol, hordenine, 5(S),15(S)‐DiHETE, and emicymarin

Eight compounds were identified that increased HDL‐C or apoA‐I protein and/or mRNA expression according to literature. It is unknown whether these compounds are also BRD4 inhibitors (Table 1; list A). Among the compounds of List A, hesperetin, equilenin, 9(S)‐HOTrE, and cymarin (Fig. 2, upper panel) were confirmed to be of natural origin as they are listed in DNP and DSM natural databases. There were six synthetic compounds that increased apoA‐I production (either measured as transcription, protein secretion, or luciferase reporter activity) and were known BRD4 inhibitors (Table 1; list B). Two of the synthetic compounds (U‐34599 and U‐51477) from list A and three of the synthetic compounds of list B (JQ1(+), Ro11‐1464 and RVX208) also increased *in vitro* apoA‐I protein secretion, besides increasing apoA‐I transcription.

**Table 1:** ApoA‐I increasing compounds according to literature review and virtual screening in the Dictionary of Natural Products (DNP) and DSM database

Compounds from literature					Compounds from the virtual screen	
List	Relation to apoA‐I	BET inhibition	Molecule	Reference	DNP	DSM
A	↑ transcription	?	9(S)‐HOTrE	van der Krieken et al. (2018)	a	a
A	↑ transcription	?	Cymarin	van der Krieken et al. (2018)	a	a
A	↑ cholesterol efflux to apoA‐I	?	BMS‐309403	Furuhashi (2007)	0	0
A	↑ cholesterol efflux to apoA‐I	?	Equilenin	Zhang et al. (2001)	a	a
A	↑ luciferase reporter activity	?	GW694481	Mirguet et al. (2012)	0	0
A	↑ cholesterol efflux to apoA‐I	?	Hesperetin	Lio (2012)	a	a
A	↑ transcription and protein secretion	?	U‐34599	Kempen et al. (2013), Princen and Kooistra (2003)	0	0
A	↑ transcription and secretion	?	U‐51477	Kempen et al. (2013), Princen and Kooistra (2003)	0	0
B	↑ luciferase reporter activity	↓	GW841819X	Chung et al. (2011)	0	0
B	↑ transcription	↓	I‐BET151	Mirguet et al. (2012)	0	0
B	↑ transcription	↓	I‐BET762	Mirguet (2013)	0	0
B	↑ transcription and protein secretion	↓	JQ1(+)	Filippakopoulos (2010), Kempen et al. (2013), McLure (2013)	0	0
B	↑ transcription and protein secretion	↓	Ro11‐1464	Zanotti et al. (2011)	0	0
B	↑ transcription and protein secretion	↓	RVX208	McNeill (2010), Gilham et al. (2016), McLure (2013)	0	0

Lists A and B were based on the outcome of the literature review and the virtual screening in the DNP and DSM databases. *Filters*: Molecular similarity compared to literature compound >0.5, present in a natural source for the virtual structural comparisons. Compounds not filtered for commercial availability.

a Data files resulting from the virtual screening in the DNP and DSM database are available upon request.

In addition, the literature search also resulted in the identification of 48 synthetic compounds that were described to bind to BRD4 (IC_50_ < 500 nM) (Table S1; list C), suggesting that they may affect apoA‐I. However, their effects on apoA‐I transcription and/or apoA‐I production have not been reported in the literature.

### *In Silico* Screening for Natural Ingredients with Potential to Increase apoA‐I

By comparison of the molecular structures within lists A and B, a structural similarity of 0.7 was found between U‐51477 and alprazolam (Table S2). Searches for structural similarity of the compounds found in the literature (list A and B) *via in silico* screening are available upon request. Among them, four natural compounds were selected based on their commercial availability (Fig. 2, lower panel).

For compound hesperetin, eriodictyol was selected (0.79 similarity); for compound equilenin hordenine was selected (0.53 similarity); for compound 9(S)‐HOTrE we selected 5(S),15(S)‐DiHETE (0.91 similarity), and for the compound cymarin we selected the structurally comparable compound emicymarin (0.81 similarity). These eight compounds were further tested for their effects on apoA‐I transcription in HepG2 cells (Fig. 2).

**Figure 3:**
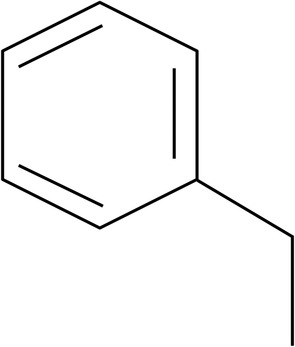
The common ethylbenzene substructure found in 60% of all BRD4 inhibitor compounds listed in Table S1 with unknown effects on apoA‐I

After determining structural similarities of these synthetic BRD4 inhibitors within list C, a common substructure (ethylbenzene moiety) was discovered in 60% of all compounds (Fig. 3). Furthermore, the DNP and DSM databases were searched for natural compounds that contained this ethylbenzene moiety. This search resulted in the identification of approximately 179 compounds with a similarity of >0.5. This data is available upon request.

### Effects of the Selected Compounds on apoA‐I mRNA Expression in HepG2 Cells

**Figure 4:**
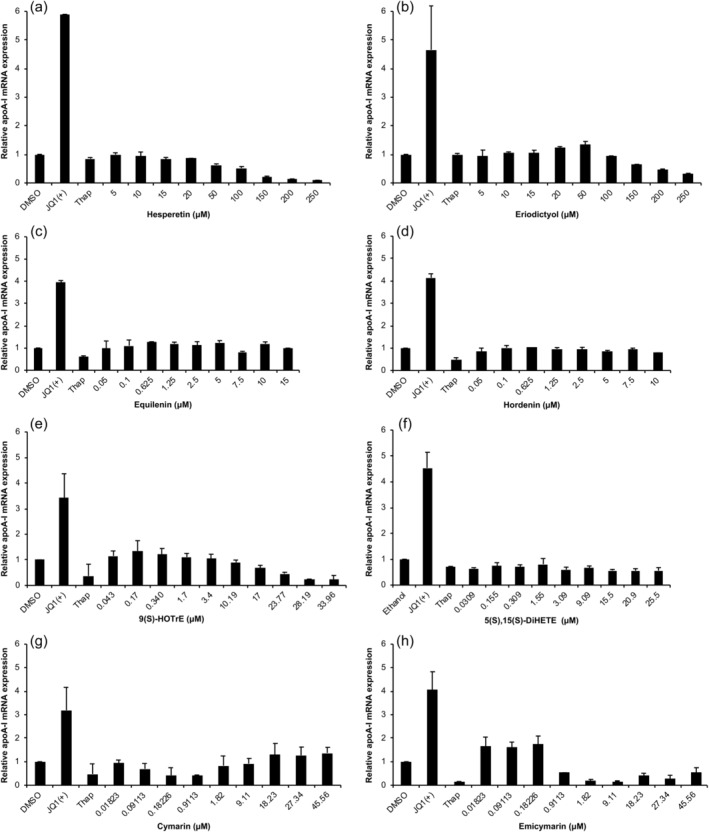
Relative apoA‐I mRNA expression in HepG2 cells treated with different doses of (a) hesperetin, (b) eriodictyol, (c) equilenin, (d) hordenine, (e) 9(S)‐HOTrE, (f) 5(S),15(S)‐DiHETE, (g) cymarin, and (h) emicymarin. Known BET inhibitor and apoA‐I increaser JQ1(+) (3 μM) was used as a positive control, whereas thapsigargin (0.01 μM) was used as a control to confirm decreased apoA‐I expression. *The data presented in panels (e) and (g) are adapted from van der Krieken et al. [23]. Compounds were tested *in duplo*; error bars indicate the SD

Addition of different doses of the known BET inhibitor RVX208 (Fig. S1) clearly increased dose dependently the production of apoA‐I mRNA. Likewise, the positive control JQ1(+) increased apoA‐I transcription in all experiments (Fig. 4), indicating that it could serve as a positive control for apoA‐I production *in vitro*. Hesperetin, did not increase apoA‐I transcription in our *in vitro* testing system. If anything, apoA‐I transcription was reduced (Fig. 4a). However, eriodictyol, the selected compound with a high similarity to hesperetin, did induce apoA‐I transcription by 35% at a dose of 50 μM (Fig. 4b). Eriodictyol in doses ranging from 100 to 250 μM decreased apoA‐I and had no effect at lower doses. Equilenin raised apoA‐I transcription in HepG2 cells, although no clear dose–response pattern was evident (Fig. 4c). Hordenine, the compound with a structure comparable to equilenin, did not affect apoA‐I transcription (Fig. 4d). 9(S)‐HOTrE increased apoA‐I transcription by 35% at a dose of 170 nM (Fig. 4e), while the structural variant of this compound 5(S),15(S)‐DiHETE, reduced apoA‐I transcription (Fig. 4f). Cymarin slightly decreased apoA‐I mRNA expression at low concentrations, but increased its expression by 37% at doses ranging from 18 to 45 μM (Fig. 4f). The structurally comparable compound emicymarin increased apoA‐I up to 77% (Fig. 4h). All compound doses were tested *in duplo* in HepG2 cells and measurements were performed *in duplo*.

## Discussion

We aimed to identify new natural compounds that increase apoA‐I transcription *via* a literature search, *in silico* screening, and *in vitro* testing. Specifically, we searched for natural BRD4 inhibiting compounds, since a growing number of studies point toward a role for BRD4 inhibitors for increasing apoA‐I transcription (Chung et al., 2011).

Based on the assumption that molecules with comparable structures interact similarly with their molecular targets (Martin et al., 2002), we examined the effects of structurally comparable compounds of hesperetin, equilenin, 9(S)‐HOTrE, and cymarin on apoA‐I transcription in HepG2 cells, namely, eriodictyol, hordenine, 5(S),15(S)‐DiHETE, and emicymarin. Of the three main structures that increased apoA‐I transcription (equilenin, 9(S)‐HOTrE, cymarin), the structurally comparable compounds eriodictyol and emicymarin increased apoA‐I transcription.

Eriodictyol, the compound that was structurally comparable to hesperetin, is a metabolite of hesperitin and both are present in oranges. We observed that, eriodictyol, but not hesperetin, induced apoA‐I transcription by 35% at a dose of 50 μM. Possibly, hesperetin needs to be converted into eriodictyol to exert its effects on apoA‐I transcription (Brand et al., 2008). If so, it would be of interest to study also effects of other metabolites of hesperetin on apoA‐I transcription. The optimal dose of eriodictyol in our study was 50 μM, which has been reported as nontoxic in various cell lines, such as macrophage mouse cells, NIH3T3 (fibroblast) cells, and HaCaT (human skin) cells (Lee et al., 2013). *In vivo* studies in mice suggested that eriodictyol is antiinflammatory (Zhu et al., 2015), while *in vitro* studies in mouse spleen cells showed that this compound has antioxidant properties (Mokdad‐Bzeouich et al., 2016). In HepG2 cells, eriodictyol increased insulin‐stimulated glucose uptake (Zhang et al., 2012) and inhibited of c‐Jun N‐terminal kinase (JNK) in macrophages (Lee et al., 2013). Since JNK is also activated during ER‐stress (Lee et al., 2011), it is of interest to examine if eriodictyol is also able to prevent ER‐stress‐induced decreases in apoA‐I transcription.

Equilenin is a naturally occurring estrogenic steroid that can be extracted from the urine of pregnant mares (Bhavnani and Stanczyk, 2014). Equilenin (10 μM) is a potent activator of the apoA‐I promoter and increased apoA‐I transcription with 140% in HepG2 cells (Zhang et al., 2001). At a dose of 10 μM, we observed that equilenin increased apoA‐I transcription by 19% in HepG2. However, the optimal dose of equilenin in our study was only 0.6 μM, which increased apoA‐I transcription by 27%. In women, estrogen replacement therapy in postmenopausal women increases plasma HDL‐C and apoA‐I concentrations (Lamon‐Fava et al., 2003). Long‐term estrogen treatment reduced the risk of mortality, myocardial infarction, or heart failure in early postmenopausal women, but not in women who start hormone therapy 5–20 years after menopause. Moreover, unwanted effects such as thromboembolic disease or breast cancer have been observed (Mosca et al., 2001). Hordenine, the structurally comparable compound of equilenin (0.53 similarity) did not influence apoA‐I transcription. Unfortunately, no other compounds with a resemblance to equilenin above 0.53 were commercially available.

9(S)‐HOTrE increased apoA‐I transcription in HepG2 cells by 35% at a dose of 170 nM (Fig. 4). 9(S)‐HOTrE is a monohydroxy polyunsaturated fatty acid found in the leaves of a plant called *Glechoma hederacea* (Kim et al., 2015). Studies in primary macrophages, suggested a possible protective role for a *Glechoma hederacea* extract on rheumatoid arthritis and osteoporosis, due to antiinflammatory actions (Hwang et al., 2014). In humans, 9(S)‐HOTrE can be produced from the essential fatty acid alpha‐linolenic acid by the action of 5‐lipoxygenase (Yandava et al., 1999). Unfortunately, the compound with a comparable structure to 9(S)‐HOTrE—5(S),15(S)‐DiHETE did not increase apoA‐I transcription.

Cymarin, a cardiac glycoside produced by the digitalis plant (Gozalpour et al., 2013), increased transcription of apoA‐I by 37% at doses ranging from 18 to 45 μM. The structurally comparable compound of cymarin, emicymarin increased apoA‐I transcription up to 77%. Emicymarin is, like cymarin, a cardenolide and cardiac glycoside. Although digoxins are used in the clinic, there is concern about its toxicity, as an *in vivo* plasma concentration above 3 μg/L could give rise to symptoms of toxicity, (Vivo et al., 2008).

The bioavailability of a compound, which was not tested in our studies, is of importance for its possible *in vivo* effects on apoA‐I transcription. In future experiments, bioavailability can therefore be addressed by using a transwell system that combines intestinal and hepatic cells. Moreover, by using the Simulator of the Human Intestinal Microbial Ecosystem (SHIME), the *in vivo* situation could be better mimicked (Van Rymenant et al., 2018). Also, more structural derivatives of the main structures that were presented could be tested. In fact, all natural compounds that have a minimum similarity above 0.5 could be examined for their effect on apoA‐I transcription. Likewise, one should investigate their effect on toxicity. Our findings further suggest that the lack of effect could be caused by the removal of a specific structural element that is essential for increasing apoA‐I transcription. It would be interesting to discover which part of a molecular structure is responsible for the increased apoA‐I transcription.

In summary, this study confirmed that the reported natural compounds equilenin, cymarin, and 9(S)‐HOTrE increase apoA‐I transcription in HepG2 cells. In addition, as predicted by *in silico* analysis, two new (structurally comparable) compounds eriodictyol and emicymarin, increased apoA‐I *in vitro*. Additional experiments are needed to confirm whether these compounds increase apoA‐I transcription by inhibiting BRD4. Additionally, it would be interesting to investigate the effects of all BRD4 inhibitors that were predicted by *in silico* modeling, on their effect on apoA‐I production, *in vitro* and *in vivo*.

## Supporting information


**Figure S1.** Relative apoA‐I mRNA expression in HepG2 cells treated with different doses of BRD4 inhibitor RVX‐208. JQ1(+) (3 μM), a known BET inhibitor and apoA‐I increaser, was used as positive control for apoA‐I expression. Error bars indicate the SD. *Dose depended increase *p* < 0.05.
**Table S1.** Results of the literature review and virtual screening in the Dictionary of Natural Products (DNP) and DSM databases of list C. CHEMBL ID and IC50 of BRD4 binding compounds are presented.
**Table S2.** Similarity of compounds from List A and B.
