# Supplementary material for: Search for Natural Compounds That Increase Apolipoprotein A‐I Transcription in HepG2 Cells: Specific Attention for BRD4 Inhibitors
Source: Lipids. 2019 Dec 8;54(11-12):687–95. doi: 10.1002/lipd.12204 (PMC7041641; doi:10.1002/lipd.12204)
Supplement: Supplementary file 1 — Figure S1. Relative apoA‐I mRNA expression in HepG2 cells treated with different doses of BRD4 inhibitor RVX‐208. JQ1(+) (3 μM), a known BET inhibitor and apoA‐I increaser, was used as positive control for apoA‐I expression. Error bars indicate the SD. *Dose depended increase p < 0.05. Table S1. Results of the literature review and virtual screening in the Dictionary of Natural Products (DNP) and DSM databases of list C. CHEMBL ID and IC50 of BRD4 binding compounds are presented. Table S2. Similarity of compounds from List A and B. [file LIPD-54-687-s001.docx]

**Supplemental data**


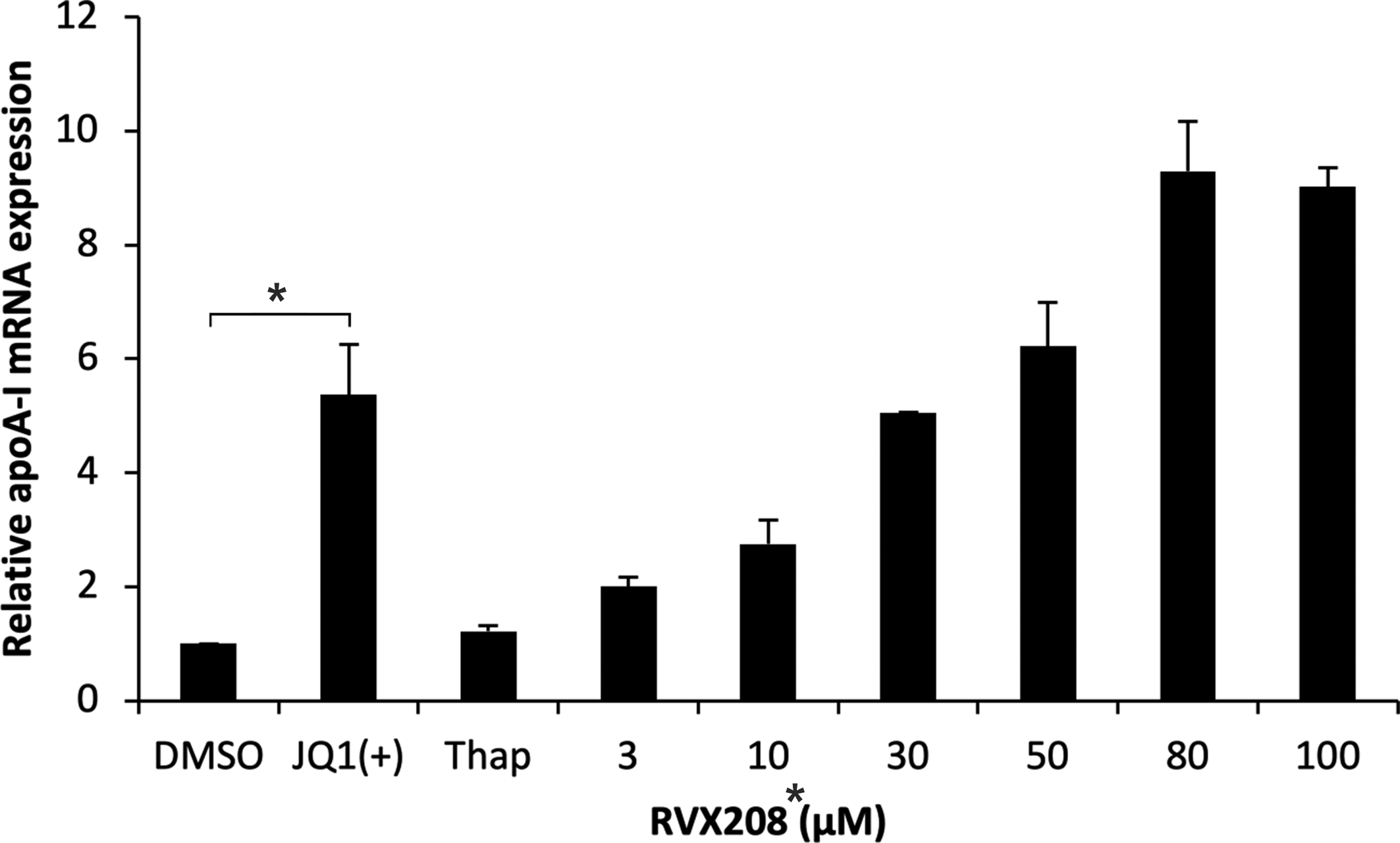


**Supplemental figure 1.** Relative apoA-I mRNA expression in HepG2 cells treated with different doses of BRD4 inhibitor RVX-208. JQ1(+) (3μM), a known BET inhibitor and apoA-I increaser, was used as positive control for apoA-I expression. Error bars indicate the standard deviation. * = Dose depended increase p<0.05.

**Supplemental table 1.** Results of the literature review and virtual screening in the Dictionary of Natural Products (DNP) and DSM databases of list C. CHEMBL ID and IC50 of BRD4 binding compounds are presented.

| CHEMBL ID | IC50 (nM) | CHEMBL ID | IC50 (nM) |
| --- | --- | --- | --- |
| CHEMBL1828979 | 1 | CHEMBL2179387 | 220 |
| CHEMBL1738926 | 16 | CHEMBL2349361 | 230 |
| CHEMBL2153434 | 16 | CHEMBL2431075 | 250 |
| CHEMBL1957266 | 24 | CHEMBL2431077 | 250 |
| CHEMBL2431090 | 26 | CHEMBL2017288 | 251 |
| CHEMBL2431078 | 29 | CHEMBL2430882 | 251 |
| CHEMBL2431079 | 33 | CHEMBL3108800 | 251 |
| CHEMBL2431091 | 36 | CHEMBL3220926 | 280 |
| CHEMBL1232461 | 36 | CHEMBL2431089 | 290 |
| CHEMBL2431080 | 42 | CHEMBL3220923 | 290 |
| CHEMBL3220922 | 50 | CHEMBL2431085 | 300 |
| CHEMBL2349340 | 59 | CHEMBL2179388 | 360 |
| CHEMBL2431081 | 72 | CHEMBL3220924 | 360 |
| CHEMBL2431082 | 73 | CHEMBL2335153 | 371 |
| CHEMBL2431076 | 74 | CHEMBL2335154 | 380 |
| CHEMBL2431093 | 86 | CHEMBL2335147 | 386 |
| CHEMBL3220925 | 100 | CHEMBL2179389 | 390 |
| CHEMBL3108801 | 158 | CHEMBL2017285 | 398 |
| CHEMBL2179385 | 180 | CHEMBL2430873 | 398 |
| CHEMBL2335155 | 180 | CHEMBL2430876 | 398 |
| CHEMBL2431074 | 190 | CHEMBL2430877 | 398 |
| CHEMBL2431094 | 190 | CHEMBL2430883 | 398 |
| CHEMBL2431073 | 200 | CHEMBL2179390 | 470 |
| CHEMBL2179386 | 220 | CHEMBL2181721 | 500 |

**Supplemental table 2.** Similarity of compounds from List A and B.

| Name | 9(S)-HOTrE | BMS-309403 | Cymarin | Equilenin | GW694481 | Hesperetin | U-34599 | U-51477 | Alaprazolam | GW841819X | I-BET151 | I-BET762 | JQ1(+) | Ro11-1464 | RVX-208 |
| --- | --- | --- | --- | --- | --- | --- | --- | --- | --- | --- | --- | --- | --- | --- | --- |
| 9(S)-HOTrE | 1 | 0.13 | 0.15 | 0.13 | 0.09 | 0.10 | 0.08 | 0.04 | 0.04 | 0.07 | 0.08 | 0.09 | 0.08 | 0.04 | 0.13 |
| BMS-309403 |  | 1 | 0.06 | 0.13 | 0.18 | 0.15 | 0.09 | 0.15 | 0.14 | 0.15 | 0.18 | 0.19 | 0.12 | 0.11 | 0.14 |
| Cymarin |  |  | 1 | 0.08 | 0.06 | 0.07 | 0.10 | 0.03 | 0.03 | 0.06 | 0.07 | 0.08 | 0.08 | 0.03 | 0.14 |
| Equilenin |  |  |  | 1 | 0.13 | 0.23 | 0.12 | 0.11 | 0.10 | 0.11 | 0.10 | 0.13 | 0.13 | 0.11 | 0.11 |
| GW694481 |  |  |  |  | 1 | 0.09 | 0.11111 | 0.13 | 0.15 | 0.17 | 0.37 | 0.13 | 0.15 | 0.19 | 0.13 |
| Hesperetin |  |  |  |  |  | 1 | 0.12 | 0.11 | 0.10 | 0.11 | 0.14 | 0.21 | 0.13 | 0.11 | 0.18 |
| U-34599 |  |  |  |  |  |  | 1 | 0.15 | 0.14 | 0.15 | 0.11 | 0.16 | 0.12 | 0.13 | 0.16 |
| U-51477 |  |  |  |  |  |  |  | 1 | 0.70 | 0.45 | 0.10 | 0.36 | 0.29 | 0.49 | 0.08 |
| Alaprazolam |  |  |  |  |  |  |  |  | 1 | 0.51 | 0.11 | 0.49 | 0.39 | 0.52 | 0.07 |
| GW841819X |  |  |  |  |  |  |  |  |  | 1 | 0.15 | 0.40 | 0.30 | 0.38 | 0.13 |
| I-BET151 |  |  |  |  |  |  |  |  |  |  | 1 | 0.18 | 0.15 | 0.16 | 0.14 |
| I-BET762 |  |  |  |  |  |  |  |  |  |  |  | 1 | 0.45 | 0.27 | 0.22 |
| JQ1(+) |  |  |  |  |  |  |  |  |  |  |  |  | 1 | 0.38 | 0.10 |
| Ro11-1464 |  |  |  |  |  |  |  |  |  |  |  |  |  | 1 | 0.075 |
| RVX-208 |  |  |  |  |  |  |  |  |  |  |  |  |  |  | 1 |
